# Effect of Type and Concentration of Carrier Material on the Encapsulation of Pomegranate Peel Using Spray Drying Method

**DOI:** 10.3390/foods10091968

**Published:** 2021-08-24

**Authors:** Katarina Šavikin, Nataša Nastić, Teodora Janković, Dubravka Bigović, Borislav Miličević, Senka Vidović, Nebojša Menković, Jelena Vladić

**Affiliations:** 1 Institute for Medicinal Plants Research “Dr. Josif Pančić”, Tadeuša Koćuška 1, 11000 Belgrade, Serbia; ksavikin@mocbilja.rs (K.Š.); tjankovic@mocbilja.rs (T.J.); dbigovic@mocbilja.rs (D.B.); nmenkovic@mocbilja.rs (N.M.); 2 Faculty of Technology, University of Novi Sad, Bulevar cara Lazara 1, 21000 Novi Sad, Serbia; nat.nastic@gmail.com (N.N.); senka.vidovic@uns.ac.rs (S.V.); 3 Department of Agriculture, Polytechnic in Požega, Vukovarska ulica 17, 34000 Požega, Croatia; borislav.milicevic@ptfos.hr

**Keywords:** *Punica granatum*, pomegranate peel, spray drying, maltodextrin, whey protein, polyphenols

## Abstract

This study aimed to establish a procedure for pomegranate peel (PP) valorization and attainment of stable extracts with preserved bioactive compounds. The technology applied was spray drying with carbohydrate-based (maltodextrin, MD) and protein-based (whey protein, WP) carrier materials in different concentrations (80, 100, and 120%). What was analyzed was the impact of the type and concentration of carrier material on the stability and quality of the final encapsulated powder. The best results were achieved when the PP extract was microencapsulated with the carbohydrate-based carrier (100%), where it had the highest encapsulation efficiency (EE) (88.63%), hygroscopicity (15.17%), and water solubility index (87.04%). The moisture content was in the range of 3.69–4.60% and 4.21–5.84% for MD and WP, respectively, indicating that both are suitable for long-term storage. It was observed that changes in carrier concentration significantly influenced most of the powders’ physicochemical properties. Microencapsulation using MD yielded a higher content of punicalin, punicalagin, gallic, and ellagic acid than those with WP. Overall results demonstrated that carbohydrate-based microencapsulation can be utilized efficiently for the protection of powder stability and phytochemical characteristics.

## 1. Introduction

Over the last decade, the production area of the pomegranate fruit (*Prunus granatum* L.) has been continuously increasing. According to Food and Agriculture Organization’s statistics [1], in 2012, the global annual production of pomegranate fruit reached approx. 1.5 million, while it was around 3 million tons in 2014 and 3.8 million tons in 2017 [2]. The processing industry of pomegranate fruits generates an enormous quantity of biodegradable pomegranate solid waste. Moreover, its disposal is time-consuming and labor-intensive and has negative impacts on environmental development and economic growth. Pomegranate peels (PPs) and internal membranes constitute around 50% of the total pomegranate weight [3]. This waste possesses functional properties that can be applied in the food, cosmetic, and pharmaceutical industries. Traditionally, this fruit by-product was used to treat various disorders of the gastrointestinal tract [4]. Functional and nutraceutical ingredients, including ellagitannins, gallotannins, flavonoids, phenolic acids, anthocyanins, complex polysaccharides, and minerals including potassium, nitrogen, calcium, phosphorus, magnesium, and sodium, have been recognized as PPs medicinal attributes. The abundance of hydrolysable tannins, specifically punicalagin, and its isomers punicalin α and β, gallic, and ellagic acid, are connected with the antioxidant activity [5], antifungal [6], anticancer [7], antidiabetic, and antineurodegenerative [8] properties of PPs.

In order to preserve bioactive compounds and to produce a food powder that allows the use of PP as a possible additive in different formulations of functional foods, it is necessary to determine the most effective encapsulation technique. Spray drying is a well-established and widely used process for creating powdered microcapsules from liquid material in one simple, easily scalable, and continuous operation by entrapping the core material with a carrier material. These advantages make it a practical candidate to be used in the food industry. However, the selection of proper carrier material in the spray drying process is crucial because it affects the drying yield, encapsulation efficiency, physicochemical properties, bioavailability, and biological activity. The commonly used carrier materials for encapsulation of food ingredients are carbohydrates and protein, or their derivatives or mixtures. Moreover, the important polysaccharides are plant-based: starch, maltodextrins (MD), corn syrup, cellulose, and amylase, with MD being the most widely applied carrier material exhibiting low viscosity, high water solubility, low cost, and neutral taste [9]. Furthermore, among the most common protein-based carrier is whey protein (WP) with low viscosity, high solubility, and good emulsifying characteristics [10].

Various studies regarding PP encapsulation by spray drying have been reported in recent years. The obtained powders were evaluated for their bioactivity, physicochemical properties, morphology, encapsulation efficiency, and application in food production [6,11,12,13]. In the study by Endo et al. [6], microparticles containing PP extract obtained by spray-drying utilizing alginate or chitosan as encapsulating agents were evaluated for their antifungal activity. Kaderides et al. [11] investigated the process of transforming PP into a food ingredient, using ultrasound extraction and encapsulation. It was determined that PP extract can improve the shelf life of hazelnut paste. In another work conducted by Kaderides and Goula [12], the authors optimized the spray drying encapsulation of PP phenolic extract using orange juice fiber powder as carrier material. Çam et al. [13] investigated the effects of core/carrier material ratio (phenolics/MD) and type of MD on product quality. The authors also evaluated the phenolics in vitro bioactivity in enriched ice cream with obtained microcapsules.

The goal of this study was to establish a feasible process that enables the conversion of pomegranate waste into a stable powder with preserved bioactive compounds. Percolation, as one of the most common ways of obtaining liquid extracts in semi-industrial and industrial conditions, was utilized for obtaining a liquid extract, which was then spray dried. Using the lowest amount of the adequate carrier, which provides powder stability and quality, is a precondition of an efficient production process. Therefore, the impact of the carriers and their concentrations on the physical properties of powders was monitored, as well as on the composition of individual phenolic compounds.

## 2. Materials and Methods

### 2.1. Chemical Reagents

Punicalagin was purchased from Sigma-Aldrich (Sternheim, Germany), Folin–Ciocalteu reagent was purchased from Merck (Darmstadt, Germany), punicalin standard was obtained from Phytolab (Vestenbergsgreuth, Germany), gallic and ellagic acid were from Extrasynthese (Genay, France), acetonitrile (HPLC grade) was Merck (Darmstadt, Germany), and ultra-pure water was prepared using a Milli-Q purification system (Millipore, Molsheim, France). Methanol (J.T. Baker, Deventer, The Netherlands) and acetonitrile (Merck, Darmstadt, Germany) were of HPLC grade.

### 2.2. Plant Material and Extraction of Phenolic Compounds

Pomegranate fruits were collected at a natural locality in village Do, Bosnia and Herzegovina, during November 2019. Peels were manually separated from the seeds, air-dried at room temperature for 4–6 days in a dark and naturally-ventilated place, and grounded using a laboratory mill. Dry plant material was grounded providing particles of 0.75–2 mm size.

Liquid extract was obtained using percolation with 70% ethanol as a solvent according to European Pharmacopoeia [14] and Grabež et al. [15]. The plant material/extract ratio was 1:5 (drug:obtained extract; *w*/*w*). Extraction was carried out at room temperature.

### 2.3. Spray Drying Process

The carrier materials were used in the liquid feed in concentrations 80, 100, and 120% compared to the dry matter of the extract (62.030 ± 0.76 mg/mL).

Concentrations of carrier materials MD (DE19.7) and WP were chosen based on conducted preliminary drying. To determine the carrier concentrations, spray drying was conducted without the addition of a carrier material and with concentrations of the carrier (10, 30, 50, and 70%). Spray drying was not successful with 0–50% carrier concentrations. With the concentration of the carrier of 70%, the obtained extract was highly hygroscopic and unstable. Therefore, 80% was the lowest concentration for obtaining the powder form. Additionally, higher concentrations (100 and 120%) were tested in order to determine the optimal (most efficient and lowest) concentration of the carrier. The amount of the carrier is expressed as a percentage of total solids in liquid extract (62.030 ± 0.76 mg/mL).

Carrier materials were dissolved in distilled water. These carrier solutions were added to the prepared extract and mixed continuously with a magnetic stirrer at a temperature of 30 °C. Such feeds were pumped into the spray drying system in mixed flow (pressure nozzle, pressure 4 bar). The prepared liquid feed was spray dried using an Anhydro spray dryer (model LAB S1, APV Anhydro AS, Soborg, Denmark). A peristaltic pump (Thermo Scientific Peristaltic pump Thermo FH100 digital, England) was used to pump the feed into the dryer. The process inlet temperatures were 120 ± 5 °C, while the outlet air temperatures were 80 ± 5 °C. The drying process was carried out at a constant pressure of 3 bar. The obtained powder was separated from the air by a cyclone. Lastly, the efficiency of powder production (expressed as the weight percentage) was determined gravimetrically as the ratio of the mass of the powder obtained after spray drying and the mass of total solids measured in the liquid feed.

### 2.4. Analysis of PPs Powder

#### 2.4.1. Moisture Content

Moisture content was determined according to the standard procedure described in the official Pharmacopeia (*Ph. Jug. IV*), by drying a sample at 105 °C until constant mass. All measurements were carried out in triplicates.

#### 2.4.2. Hygroscopicity

Hygroscopicity measurements were performed using the method described by Vladić et al. [16]. Samples of each powder (approximately 1 g) were placed at 25 °C in an airtight container or desiccators filled with sodium chloride saturated solution (70% relative humidity). The hygroscopicity was monitored after 24, 48, and 5 days. Hygroscopicity was expressed as a gram of absorbed water per 100 g of dry extract powder. All measurements were carried out in triplicates.

#### 2.4.3. Rehydration

The time needed for the powder to completely rehydrate (expressed in seconds) was determined by adding 2 g of dry extract into 50 mL distilled water at room temperature. The mixture of powder and water was mixed via a magnetic stirrer in a glass flask.

#### 2.4.4. Bulk Density

Bulk density was measured by determining the volume occupied by the dry extract mass. Powder (1 g) was freely added into a 20 mL graduated glass cylinder and exposed to vibration for 2 min. Bulk density was calculated from the difference of the empty glass cylinder and the mass of the glass cylinder with powder and expressed as mg of powder per mL. All measurements were carried out in triplicates.

#### 2.4.5. Water Solubility Index and Water Absorption Index

Determination of the water solubility index (WSI) and water absorption index (WAI) was achieved according to a previously described method [17]. Measures of 1.5 g of powder and 15 mL water were vigorously mixed in a 50 mL centrifuge tube; the mixture was incubated in a water bath at 30 °C for 30 min, and centrifuged at 3000 rpm for 15 min. The supernatant was collected in a pre-weighed Petri dish, and the residue was weighed after oven drying at 105 °C overnight. WSI was calculated as the ratio of the mass of dried supernatant and the mass of the dry sample. WAI was calculated as the mass of solid pellets remaining after the elimination of the supernatant divided by the mass of the dry sample. All measurements were carried out in triplicates.

### 2.5. HPLC Analysis

HPLC analysis was performed according to the modified method by Kam et al. [18]. The analyses were carried out on the Agilent 1200 RR HPLC instrument with the DAD detector (Agilent, Waldbronn, Germany) using the reverse phase Zorbax SB-C18 (Agilent) analytical column (150 mm × 4.6 mm i.d.; 5 µm particle size). The mobile phase consisted of solvent A (1% v/v solution of orthophosphoric acid in water) and solvent B (acetonitrile). Separation was achieved according to the following scheme: 0–5 min, 98–90% A; 5–15 min, 90% A; 15–20 min, 90–85% A; 20–25 min, 85–70% A; 25–30 min, 70–40% A; 30–34 min, 40–0% A. Detection wavelengths were set at 260, 280, 320, 360, and 380 nm, and the flow rate was 1 mL/min. The injection volume was 3 μL, while the column temperature was maintained at 25 °C. The amounts of the compounds in investigated extracts were calculated using calibration curves for standard compounds (gallic acid, ellagic acid, punicalin, and punicalagin). Results were expressed as milligram per gram of powder.

### 2.6. Statistical Analysis

All analyses were carried out in triplicate and the results were expressed as means ± standard deviation (SD). Mean values were considered significantly different at *p* < 0.05 confidence level, after the performance of the one-way ANOVA statistical analysis followed by Tukey test using the web-based open-access tool Astatsa Online Web Statistical Calculators (Navendu Vasavada, astatsa.com; accessed on 4 February 2021).

## 3. Results

### 3.1. Encapsulation Efficiency

**Figure 1:**
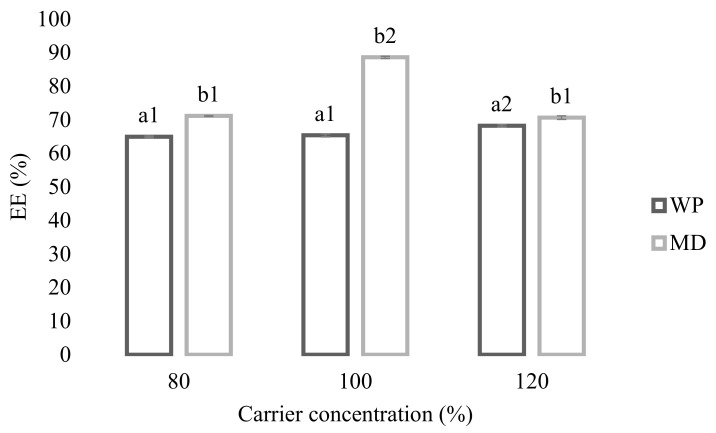
Efficiency of the spray drying process. Different letters indicate significant difference between WP and MD powders (*p* < 0.05); different numbers indicate significant difference between carrier concentrations (*p* < 0.05).

The production of final powder particles with the best properties is the main objective of the encapsulation process. Hence, it should be performed by considering the critical influencing factors on the final powder properties that might be listed as feed flow rates, inlet air temperature, carrier materials ratio/type, core ratio/type, etc. EE is an important parameter in food powders production by spray drying related to the feed properties and spray drying process conditions [19]. A minimum of 50% of EE is deemed successful in laboratory and pilot-scale spray dryers [20]. In this study, the effects of three concentrations of carrier materials on the EE were investigated. EE of the various carrier formulation used in this research study is shown in Figure 1. EE was positively influenced by WP concentration, being in the range from 64.95 to 68.23% when the WP concentration increased from 80 to 120%, while the highest EE values were observed in the microparticles produced by using 100% MD as the carrier material (88.63%). It is apparent that the presence of WP in the formulations negatively affected their EE in comparison to MD. The powders prepared with the addition of MD showed relatively higher EE than the WP used, regardless of concentration. This trend was similar to that reported by Jokić et al. [21] for the encapsulation of cocoa bean shell extracts using MD and WP as carrier materials. Another study reported similar results for microparticles containing PP extract by the spray drying technique, utilizing alginate (81.9%) and chitosan (74.7%) as the carrier materials [6]. Kaderides et al. [11] encapsulated PP extract using common carrier materials (MD, WP, skim milk powder, and gum arabic) and reported that the maximum EE of 98.64% was obtained using a mixture of MD/WP (50:50) as the carrier material and by co-current drying in a spray dryer of a lower capacity.

### 3.2. Moisture Content and Hygroscopicity

The study of moisture content is of fundamental importance to the powders, related to the powder stability, flowability, drying efficiency, stickiness, oxidation of bioactive agents, and microbial growth. The moisture content of the PP powders varied between 3.69% and 5.84%, with less than 6% being in accordance with the moisture content requirement of a food powder (4–6%) suitable for long-term storage [22]. Table 1 shows that higher moisture content was obtained when lower WP and higher MD concentrations were used individually, showing similar moisture content at the concentration of 100%. WP showed a comparatively higher moisture content (5.84%) to MD (3.69%), probably because of the better water holding capacity of proteins [23]. An increase in microcapsules moisture content with increased WP concentration might also be caused because WP was not crystallized upon drying and water removal [24]. Conversely, the higher concentration of MD caused a significant reduction in the moisture content that resulted in a good carrier formation. These results were lower than those of spray-dried PP microcapsules obtained using edible orange by-product fiber as the carrier material (6.34–12.80%) [12], but similar to those of PP microcapsules spray-dried using MD/WP (50:50) [11] and modified starch (Capsul) [25].

One of the main characteristics of powder stability is also hygroscopicity. GEA Niro [26] provided the powder hygroscopicity classification: hygroscopic (15–20%), slightly hygroscopic (10–15%), and non-hygroscopic powder (<10%). The hygroscopicity of PP powders was monitored after 1, 2, and 5 days. The results showed that the PP powder had low hygroscopicity at the beginning of the storage, and that it slightly increased over storage time using both carrier materials (Table 2). Hygroscopicity values reported in our research follow the same trend of increase as previously observed by Jokić et al. [21] for powders of cocoa bean shells. PP powders with MD as the carrier material exhibited significantly higher hygroscopicity than powders with WP as the carrier. According to Pérez-Alonso et al. [27], the proteins involved in powder particles modified the balance of hydrophilic/hydrophobic sites adsorbed at the interface, decreasing the water adsorption property. Clearly, by increasing the concentration of WP carrier material from 80 to 120%, the hygroscopicity in the microcapsules decreased; thus, the highest values were seen at 80% concentration (12.24%). In addition, Tonon et al. [28] found that lower moisture contents of powders had the opposite effect on the hygroscopicity probably due to the relation between the ability to absorb moisture and the water gradient concentration between the powder and the atmosphere. This is in accordance with the results for WP powders in this study. On the other side, Ahmed et al. [29] stated that there was no direct relationship between hygroscopicity and moisture content of sweet potato powder spray-dried by different carrier materials. However, the results showed that PP obtained with MD possessed the lowest content of moisture and lowest hygroscopicity. Consequently, it is not possible to generalize the moisture-hygroscopicity relationship for all products. Therefore, each product demands an individualized approach.

### 3.3. Bulk Density

The bulk density is an important food powder parameter for storage, processing, packaging, and distribution that depends on the powder size, shape, surface properties, and particle size. Smooth and uniform powder has a higher bulk density, indicating a lower amount of air between powder cavities, thus preventing lipid oxidation and increasing storage stability [30]. The bulk density of the PP extract was affected by different carrier materials and varied from 175.29 to 344.51 mg/mL (Table 3). The WP powders at higher concentrations exhibited higher bulk density in comparison to formulations containing MD, while the 100% MD powder displayed the lowest bulk density among other WP and MD powders. Goula and Adamopoulos [31] and Pieczykolan and Kurek [32] explained that differences in bulk density with rising concentrations of carrier material could be attributed to the high molecular weight of the microcapsules/carrier material. The higher bulk density values of powders containing WP as the carrier material might be due to the small particles inserted in the interparticle spaces. Conversely, MD powder particles might be stuck together, resulting in the formation of free air spaces in the powder. The values from this study were similar to those reported by Shishir et al. [33] for spray-dried powders from pink guava puree using MD as the carrier material. MD is considered a skin-forming material, producing low dense particles due to crust formation and trapped air inside the particles [34]. However, Kaderides and Goula [12] reported higher values of bulk density during spray drying of PP using edible orange by-product fiber as the carrier material.

### 3.4. Water Absorption Index, Water Solubility Index, and Rehydration

The rehydration properties of the microparticles were evaluated in terms of WAI, WSI, and rehydration time. WAI and WSI are inversely related and indicate the amount of water immobilized by the samples and the amount of soluble solids present in the product, respectively. WSI values were between ranges of 63.34–87.04% (Table 4). These values are similar to those obtained by Navarro-Flores et al. [35] for the encapsulation of *Crotalaria longirostrata* leaves, and higher than those obtained by Vidović et al. [36] for the encapsulation of aronia fruit dust (average of 57.2%), but lower than WSI obtained for white horehound powder (92.19%) [37]. By comparing the WAIs of the powders, it can be inferred that the microcapsules prepared with WP had significantly higher adsorption in comparison to MD, with the highest being in 80% WP powder, indicating that it is more likely to be affected by humidity and less stable during storage. On the other hand, microcapsules produced with MD had significantly more WSI (average of 85.16%) than WP (average of 66.10%), and increasing MD concentration from 80 to 120% improved the WSI percentage. This indicates that MD powders could be easily and effectively reconstituted in water, easily incorporated, and evenly distributed into products. An increase of WSI with an increase of MD mass fraction was also reported in the study by Vidović et al. [36] for spray drying of aronia fruit dust.

In general, complete powder rehydration is required in order to exploit its functionality. This quality attribute of food powders should take place within a short time period, minimizing manufacturing time and production costs, and being practical and economical for most industrial processes. In this study, rehydration time tends to rise with the addition of WP (Table 4). Conversely, using higher MD concentrations reduced the time required for rehydration. This trend could be associated with low moisture content and, thus, the low powder stickiness, and consequently the higher surface area in contact with water [31]. Similarly, Aragüez-Fortes et al. [38] reported that guava powder was more soluble with a higher MD concentration. The rehydration time for the spray-dried PP powder with MD varied from 90 to 145 s, while the powder containing WP presented lower values (42–100 s). Similar trends in the behavior of powders have been reported by Jokić et al. [21], where higher rehydration time is reported for MD than WP for the spray-dried powder of cocoa bean shells extract.

### 3.5. HPLC Analysis of Polyphenolic Compounds

**Figure 2:**
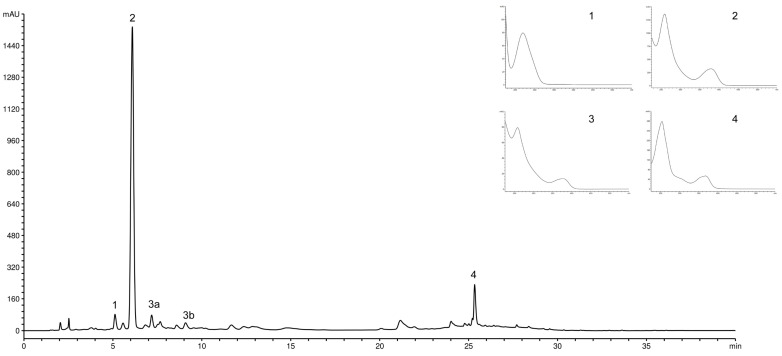
HPLC chromatogram of the pomegranate peel powder produced with 80% maltodextrin recorded at 260 nm is provided, with the spectrum of identified compounds. Peaks: 1, gallic acid; 2, punicalin; 3a, ɑ-punicalagin; 3b, β-punicalagin; 4, ellagic acid.

The effect of the type and concentration of carrier material on the content of major compounds in the PP microcapsules is shown in Table 5. The dominant compound was punicalin, followed by punicalagin, gallic, and ellagic acid (Figure 2). Similar findings were reported by Çam et al. [13], who indicated that these types of compounds were the main phenolics of spray-dried PP powder using MD. The content of individual polyphenolic compounds of the microcapsules produced with MD was higher than those with WP, which is probably due to a better protective effect of MD on the phenolics of the PP extract. It seems that PP phenolic compounds have a higher binding capacity for MD as polysaccharides than for protein-based WP. Polyphenol-carbohydrate and polyphenol-protein interactions are dependent on the structure of polyphenols, their molecular weight, and the number of hydroxyl groups [39] According to Najafi et al. [40], the compound distribution into the microcapsules shell and their displacement to the surface are directly affected by the molecular dimensions of carrier materials. A significant reduction (*p* > 0.05) in polyphenolic compounds content was noticed for powders obtained using a higher concentration of each carrier. The content of punicalin in 80% MD wall material was determined as 169.91 mg/g, which was 1.3-fold higher than in 120% MD as measured in the present study. Nogueira et al. [41] confirmed the dependence of phenolics retention upon carrier type and its concentration. The authors observed that the formulations containing the Capsul carrier exerted the greatest influence on punicalagin retention, similar to MD, while the gum arabic was the carrier that contributed less to its retention. Similar results were reported by Bustamante et al. [25] for encapsulated PP extract prepared by modified starch (Capsul).

## 4. Conclusions

This research highlights the use of spray drying as an efficient technique for obtaining microparticles and preserving the bioactive compounds of PPs. Microparticles obtained with MD demonstrated the most adequate preservation of polyphenolic compounds for PP after spray drying. The final encapsulated powder features were influenced by both type and concentration of the carrier material. The highest EE (88.63%), hygroscopicity (15.17%), and water solubility index (87.04%) were achieved when the PP extract was microencapsulated with the carbohydrate-based carrier (100%). Moisture content of MD and WP powders varied between 3.69 and 5.84%, both being suitable for long-term storage. MD was a more suitable carrier than WP for the encapsulation of PP bioactive compounds, improving their bioavailability and functionality. Forthcoming studies should be directed towards the evaluation of the powder bioavailability by carrying out in vitro and in vivo tests, the evaluation of powders on the basis of rheological and sensory properties, and the incorporation of PP powders into functional products for human consumption.

## Figures and Tables

**Table 1:** Moisture content of PP powders.

Carrier Concentration (%)	Moisture Content (%)	
	WP	MD
80	4.27 ± 0.08 ^a1^	4.60 ± 0.09 ^b1^
100	4.21 ± 0.03 ^a1^	4.29 ± 0.07 ^a2^
120	5.84 ± 0.11 ^a2^	3.69 ± 0.04 ^b3^

Different letters within the same row and different numbers within the same column indicate a significant difference between samples at *p* < 0.05.

**Table 2:** Effect of storage time on the hygroscopicity (%) of PP powders.

Time		24 h		48 h		5 Days	
Carrier		WP	MD	WP	MD	WP	MD
Carrier concentration (%)	80	10.75 ± 0.08 ^a1^	12.17 ± 0.04 ^a1^	11.98 ± 0.02 ^b1^	13.92 ± 0.04 ^b1^	12.24 ± 0.02 ^c1^	14.89 ± 0.06 ^c1^
	100	10.72 ± 0.04 ^a1^	13.02 ± 0.06 ^a2^	11.14 ± 0.08 ^b2^	14.52 ± 0.09 ^b2^	11.62 ± 0.09 ^c2^	15.17 ± 0.02 ^c2^
	120	8.07 ± 0.06 ^a2^	12.67 ± 0.07 ^a3^	9.09 ± 0.09 ^b3^	13.90 ± 0.08 ^b1^	9.17 ± 0.08 ^b3^	14.30 ± 0.03 ^c3^

Different letters within the same row (the same carrier) and different numbers within the same column indicate a significant difference between samples at *p* < 0.05.

**Table 3:** Bulk density of PP powders.

Carrier Concentration (%)	Bulk Density (mg/mL)	
	WP	MD
80	180.06 ± 2.14 ^a1^	218.72 ± 4.63 ^b1^
100	198.90 ± 3.31 ^a1^	175.29 ± 4.04 ^b2^
120	344.51 ± 6.41 ^a2^	230.87 ± 6.28 ^b1^

Different letters within the same row and different numbers within the same column indicate a significant difference between samples at *p* < 0.05.

**Table 4:** Water absorption index, water solubility index, and rehydration time of PP powders.

		WAI (%)		WSI (%)		Rehydration Time (s)	
Carrier		WP	MD	WP	MD	WP	MD
Carrier concentration (%)	80	28.79 ± 0.12 ^a1^	11.12 ± 0.09 ^b1^	65.34 ± 0.51 ^a1^	83.46 ± 0.69 ^b1^	42 ± 1.3 ^a1^	145 ± 3.9 ^b1^
	100	23.47 ± 0.2 ^a2^	5.37 ± 0.11 ^b2^	69.63 ± 0.63 ^a2^	84.98 ± 0.83 ^b1^	46 ± 2.1 ^a2^	92 ± 2.6 ^b2^
	120	28.44 ± 0.15 ^a1^	8.69 ± 0.08 ^b3^	63.34 ± 0.72 ^a3^	87.04 ± 0.92 ^b2^	100 ± 4.8 ^a3^	90 ± 1.8 ^b2^

Different letters within the same row and different numbers within the same column indicate a significant difference between samples at *p* < 0.05.

**Table 5:** Polyphenolic compound content of PP powders.

Sample	Gallic Acid (mg/g Powder)	Punicalin (mg/g Powder)	Punicalagin α + β (mg/g Powder)	Ellagic Acid (mg/g Powder)
80% MD	6.33 ± 0.56 ^12^	169.91 ± 1.90 ^1^	8.92 ± 0.87 ^1^	7.46 ± 0.28 ^1^
100% MD	5.55 ± 0.38 ^23^	144.69 ± 2.53 ^2^	7.03 ± 0.59 ^2^	6.77 ± 0.15 ^12^
120% MD	4.79 ± 0.47 ^3^	128.44 ± 2.11 ^3^	6.65 ± 0.38 ^2^	6.52 ± 0.44 ^2^
80% WP	5.76 ± 0.24 ^1^	143.36 ± 3.23 ^1^	6.3 ± 0.67 ^1^	4.73 ± 0.21 ^1^
100% WP	4.32 ± 0.19 ^2^	123.52 ± 1.78 ^2^	5.77 ± 0.72 ^1^	4.03 ± 0.62 ^1^
120% WP	3.78 ± 0.32 ^2^	105.37 ± 2.17 ^3^	5.31 ± 0.34 ^1^	3.99 ± 0.33 ^1^

Different numbers within a column (the same carrier) indicate a significant difference between samples at *p* < 0.05.

## Data Availability

Not application.
